# The ups and downs of global motion perception: a paradoxical advantage for smaller stimuli in the aging visual system

**DOI:** 10.3389/fnagi.2014.00199

**Published:** 2014-08-08

**Authors:** Claire V. Hutchinson, Tim Ledgeway, Harriet A. Allen

**Affiliations:** ^1^College of Medicine, Biological Sciences and Psychology, School of Psychology, University of LeicesterLeicester, UK; ^2^School of Psychology, University of NottinghamNottingham, UK

**Keywords:** vision, global motion, age, aperture size, dot density

## Abstract

Recent evidence suggests that normal aging is typically accompanied by impairment in the ability to perceive the global (overall) motion of visual objects in the world. The purpose of this study was to examine the interplay between age-related changes in the ability to perceive translational global motion (up vs. down) and important factors such as the spatial extent (size) over which movement occurs and how cluttered the moving elements are (density). We used random dot kinematograms (RDKs) and measured motion coherence thresholds (% signal elements required to reliably discriminate global direction) for young and older adults. We did so as a function of the number and density of local signal elements, and the aperture area in which they were displayed. We found that older adults’ performance was relatively unaffected by changes in aperture size, the number and density of local elements in the display. In young adults, performance was also insensitive to element number and density but was modulated markedly by display size, such that motion coherence thresholds decreased as aperture area increased (participants required fewer local elements to move coherently to determine the overall image direction). With the smallest apertures tested, young participants’ motion coherence thresholds were considerably higher (~1.5 times worse) than those of their older counterparts. Therefore, when RDK size is relatively small, older participants were actually better than young participants at processing global motion. These findings suggest that the normal (disease-free) aging process does not lead to a general decline in perceptual ability and in some cases may be visually advantageous. The results have important implications for the understanding of the consequences of aging on visual function and a number of potential explanations are explored. These include age-related changes in spatial summation, reduced cortical inhibition, neural blur and attentional resource allocation.

## Introduction

The ability to effectively discriminate the movement of objects in the world around us is a fundamental part of visual perception. It requires us to be able to decode a constantly, and often rapidly, updated retinal image. These changing patterns of visual information that impinge on our retinas are generated, not only by multiple objects moving independently in the environment, often along different trajectories and at different velocities, but also by our own self-motion relative to stationary objects as we navigate our way through space.

Age-related declines in the ability to accurately decode the moving world have often been reported, particularly with respect to global motion patterns in which individual (“local”) elements contribute to the overall pattern motion of a larger (“global”) stimulus. The majority of studies that have investigated age-related deficits in global motion perception have employed random dot kinematograms (RDKs) in which local elements, or dots, move along a translational trajectory (i.e., left or right; up or down). However, these studies have produced mixed results. Whilst some show a deleterious effect of age for translational global motion perception (e.g., Trick and Silverman, 1991; Wojciechowski et al., 1995; Tran et al., 1998; Billino et al., 2008), others have failed to find differences (e.g., Gilmore et al., 1992) between young and old participants’ performance. Other studies still have failed to find age-related deficits under some conditions but found impaired performance for older participants under others (e.g., Atchley and Andersen, 1998; Snowden and Kavanagh, 2006; Allen et al., 2010). It is likely therefore that the degree of age-related deficits in global motion perception is heavily dependent on the precise parameters of the RDK patterns used. For example, we (Allen et al., 2010) have recently demonstrated that when high contrast (30% Michelson) dots move at a speed of 5.6 deg/s, older and younger adults’ motion discrimination performance are equivalent. However when dots were lower contrast (but still supra-threshold, <~4 %) we found elevated motion coherence thresholds for older, compared to younger adults. These findings demonstrate the importance of contrast sensitivity in global motion perception.

The relative effects of other factors such as aperture area (overall stimulus size) and dot number/density on age-related global motion deficits are presently unclear, despite these being strong influences on performance in younger adults (see Hutchinson et al., 2012). As the size of a stimulus increases, detection and discrimination thresholds typically decrease (e.g., Lappin and Bell, 1976; Anderson and Burr, 1991; Watson and Turano, 1995; Hutchinson and Ledgeway, 2010). This is due to spatial summation whereby the larger stimuli contain more contrast energy and are thus more detectable. In older adults, however, there appears to be a further advantage for large, high contrast, moving gratings compared to younger adults. Betts et al. (2005) compared older and younger adults with high and low contrast sine wave gratings that subtended either 0.7 or 5 degrees. Whilst older adults required longer stimulus presentation times to discriminate motion direction than younger adults, with large high contrast stimuli they needed shorter durations than younger adults. In a similar task, with counter-phasing Gabor stimuli, matched for individual contrast sensitivity, younger adults showed less summation at higher contrasts than lower contrasts whereas older adults continued to show summation even at high contrasts. It has been suggested by some that these age-related differences may be due to differences in spatial suppression (Betts et al., 2005) in extrastriate cortical area V5/MT (Glasser and Tadin, 2010) or contrast sensitivity (Aaen-Stockdale et al., 2009). One might expect a similar advantage for large global motion RDK patterns, but given that the effects of aging on summation area are not necessarily consistent between different stimuli (e.g., Dannheim and Drance, 1971; Schefrin et al., 1998) this is currently an unresolved issue.

Although studies suggest that increasing stimulus area may have differential effects in young and older adults for simple motion, none have investigated age-related differences in the context of global motion perception. In addition, the effects of age on performance for encoding other RDK parameters such as the number or density of individual elements (local dots) in the display are presently unknown. In the context of studies that have not specifically studied the effects of aging on motion vision, Barlow and Tripathy (1997) have shown, in a small sample of adults, that motion coherence thresholds decrease with increasing stimulus area. They also showed a nominal effect of local element density, although this only translated to an improvement of less than 20% over a 64-fold increase in local element density (1.7–111 dots/deg^2^). Others (e.g., Williams and Sekuler, 1984) have found no effect of local element density on the probability of perceiving unidirectional flow in RDKs, although Eagle and Rogers (1996) have shown that increasing local element density may lead to an increase in D_max_ (the maximum displacement for reliable direction identification). Dakin et al. (2005) have suggested that the number of local elements in the display is the principal factor that determines performance for discriminating global motion. Using an equivalent noise paradigm, they showed that local and global limits on direction integration are determined by the number of local elements in the display, irrespective of their density or indeed the size of the aperture in which they are displayed. Even if summation areas do not change with age, it is possible that the mechanism that serves to integrate over local motion elements is different, or at least subject to different constraints, in younger and older adults. Mapstone et al. (2008), for example, found no differences in older adults’ heading discrimination with moving stimuli of different sizes unless a conflicting pattern was presented in the periphery.

To address these issues we conducted three experiments to investigate how varying the number and density of local signal elements, and the aperture area in which they are displayed affects performance for discriminating the global direction of translational RDKs in young and older adults.

## Materials and methods

### Participants

Twelve young (mean age = 21.4 years, SD = 2.95) and 10 older (mean age = 72.9 years, SD = 2.33) participants took part in Experiment 1. 14 younger (mean age = 21.18 years, SD = 2.89) and 11 older (mean age = 72.82 years, SD = 2.23) participants took part in Experiment 2. 9 younger (mean age = 22.00 years, SD = 2.84) and 9 older (mean age = 71.33 years, SD = 4.21) participants took part in Experiment 3. All had normal or corrected-to-normal visual acuity and normal binocular vision. Different groups of participants took part in each experiment. Older participants were screened for major head injuries and dementia using the mini mental state examination (Folstein et al., 1983). All experimental methods adhered to the tenets of the Declaration of Helsinki and were approved by the relevant institutional ethics committees.

### Apparatus and stimuli

**Figure 1:**
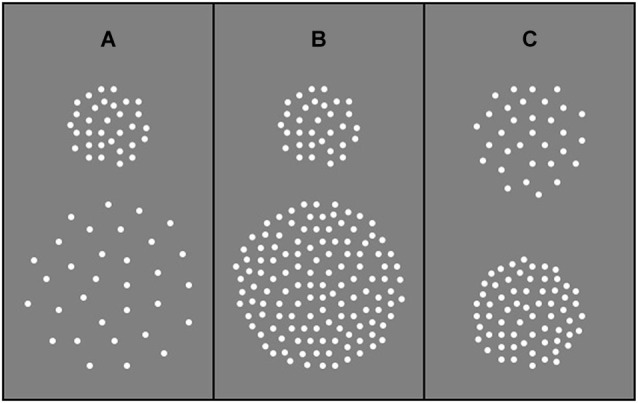
**Examples of stimulus composition. (A)** Experiment 1: Dot number remained constant irrespective of aperture size such that dot density decreased as aperture size increased. **(B)** Experiment 2: Dot number increased with increasing aperture size such that dot density remained constant. **(C)** Experiment 3: Dot number varied and aperture size remained constant such that increasing dot number led to an increase in dot density.

Stimuli were generated using a *Macintosh G4* and presented on a* P255f Professional* monitor (refresh rate 75 Hz) that was gamma-corrected with the aid of internal look-up tables. Stimuli were RDKs depicting translational (up vs. down) motion. Dots (0.47 deg diameter) were high contrast (30% Michelson) and were presented in a central aperture on a homogenous “gray” background (background luminance 64.72 cd/m^2^). Viewing distance was 92 cm. Each RDK was generated immediately prior to its presentation and was composed of a sequence of 8 images (each 53.3 ms), which when presented consecutively produced continuous motion lasting 426.7 ms. At the beginning of each motion sequence, the position of each dot was randomly assigned. On subsequent frames, each dot was shifted by 0.3 deg, resulting in a drift speed of 5.7 deg/s. When a dot dropped off the edge of the circular display window it was immediately re-plotted in a random spatial position within the window. In Experiment 1, aperture area varied in the range 28–227 deg^2^ but dot number remained constant (64 dots), such that a two-fold increase in aperture area corresponded to a two-fold decrease in dot density (in the range 2.26–0.28 dots/deg^2^). In Experiment 2, aperture area varied from 14 to 227 deg^2^ but dot density remained constant (1.13 dots/deg^2^) across experimental conditions, such that a two-fold increase in aperture area corresponded to an equivalent increase in dot number (in the range 16–256 dots). In Experiment 3, aperture area remained constant at 113 deg^2^ and two dot densities (0.44 and 1.13 dots/deg^2^) were presented. A stimulus schematic for each experiment is shown in Figure 1.

### Procedure

The global motion coherence level of the stimulus was manipulated by constraining a fixed proportion of “signal” dots on each image update to move coherently along a translational trajectory (either upwards or downwards on each trial with equal probability). The remainder (“noise” dots) moved in random directions. Global motion thresholds were measured monocularly using a single interval, forced choice, direction discrimination procedure. The participants’ task was to identify whether the global motion was upwards or downwards. The order of testing was randomized. Participants completed at least four 3-down, 1-up adaptive staircases (Edwards and Badcock, 1995) that varied the proportion of signal dots present on each trial, according to the observer’s recent response history. The staircase terminated after eight reversals and thresholds (79% correct performance) were taken as the mean of the last six reversals.

## Results

**Figure 2:**
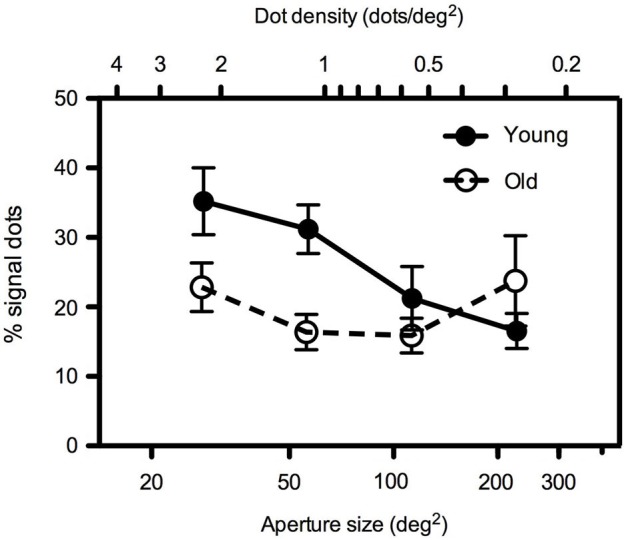
**Experiment 1: Mean global motion coherence thresholds (% signal dots supporting 79% correct direction discrimination performance) for “young” and “old” participants as a function of aperture area.** Dot number remained constant (64 dots) such that a two-fold increase in aperture area (in the range 28–227 deg^2^) corresponded to a two-fold decrease in dot density (in the range 2.26–0.28 dots/deg^2^). Error bars = ±1 S.E.M.

In Experiment 1, dot number remained constant at 64 dots such that dot density decreased as aperture size increased. Findings are presented in Figure 2 which shows mean global motion coherence thresholds (% of signal dots required for 79% correct direction discrimination performance) for younger and older participants as a function of aperture size. Younger participants’ performance was more markedly affected by changes in aperture size/dot density than that of older participants. A 2 (age) × 4 (aperture size) mixed analysis of variance (ANOVA) showed that there was a significant interaction between age and aperture size [*F*_(1.535, 30.709)_ = 4.568, *p* < 0.05]. Closer inspection of the data using one-way ANOVA, performed separately for each age group, confirmed that younger participants’ motion coherence thresholds decreased significantly as aperture size increased [*F*_(3, 47)_ = 5.401, *p* < 0.005] whereas older participants’ performance remained relatively immune to changes in the spatial aperture [*F*_(3,39)_ = 1.019, ns]. Furthermore, for conditions in which the aperture size was relatively large (≥~113 deg^2^), there was no significant difference between younger and older participants’ performance. However at smaller aperture sizes (≤~57 deg^2^), younger participants exhibited higher motion coherence thresholds (performance was worse) than older participants (see Table 1 for further details of *t*-test pairwise comparisons).

**Table 1:** ***t*-test results comparing motion coherence thresholds at each aperture size in younger and older participants in Experiment 1**.

**Aperture area (deg^2^)**	***t***	***df***	***p***
**28.270**	2.00	20	0.059
**56.750**	2.69	20	0.014
**113.10**	1.35	20	0.191
**226.98**	−1.16	20	0.261

**Figure 3:**
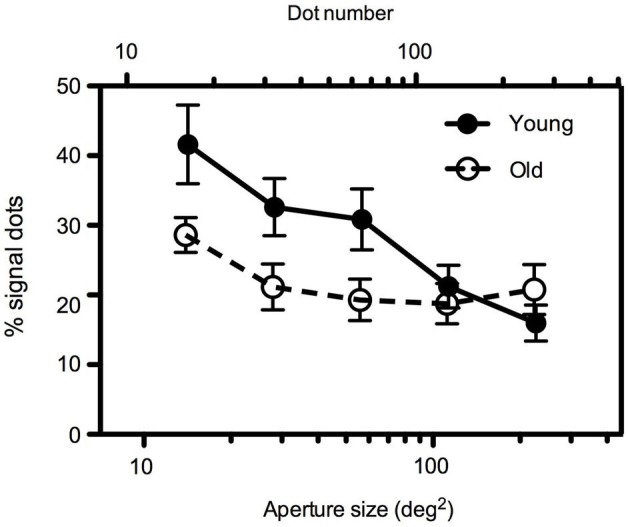
**Experiment 2: Mean global motion coherence thresholds for “young” and “old” participants as a function of aperture area.** Dot density remained constant (1.13 dots/deg^2^) across conditions such that a two-fold increase in aperture area (in the range 14–227 deg^2^) corresponded to an equivalent increase in dot number (in the range 16–256 dots). Error bars = ±1 S.E.M.

To separate the effects of aperture size and dot density, in Experiment 2, dot number increased with increasing aperture area such that dot density remained constant across conditions at 1.13 dots/deg^2^. Mean global motion coherence for younger and older participants as a function of aperture size are shown in Figure 3. Even when dot density remained constant the findings were comparable to those in Experiment 1. A 2 (age) × 5 (aperture size) mixed ANOVA again showed a significant interaction between age and aperture size [*F*_(4,88)_ = 7.945, *p* < 0.0001]. For younger participants, motion coherence thresholds decreased significantly as aperture size increased [*F*_(4,64)_ = 6.042, *p* < 0.0001]. For older participants performance remained relatively consistent irrespective of the spatial extent of the image [*F*_(4,54)_ = 1.956, ns]. For large apertures (≥~113 deg^2^), there was no significant difference between younger and older participants’ performance, but for smaller aperture sizes younger participants motion coherence thresholds were higher than older participants (see Table 2 for further details).

**Table 2:** ***t*-test results comparing motion coherence thresholds at each aperture size in younger and older participants in Experiment 2**.

**Aperture area (deg^2^)**	***t***	***df***	***p***
**14.186**	2.11	22	0.051
**28.270**	2.27	22	0.034
**56.750**	2.11	22	0.046
**113.10**	0.62	22	0.540
**226.98**	−1.11	22	0.279

**Figure 4:**
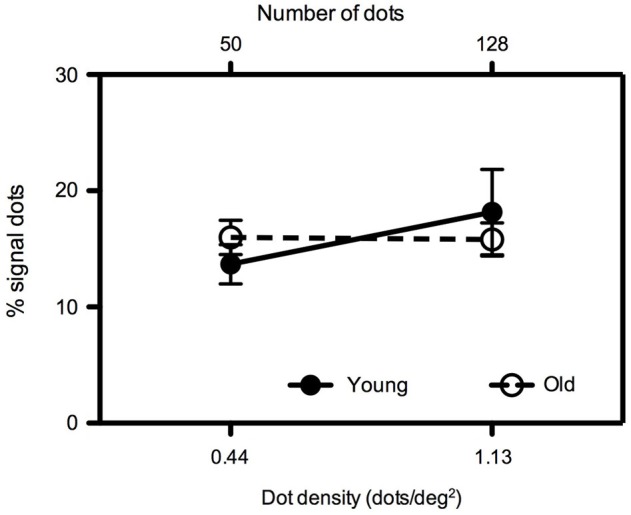
**Experiment 3: Mean global motion coherence thresholds for “young” and “old” participants at two dot densities (0.44 and 1.13 dots/deg^2^) with an aperture size of 113 deg^2^.** Error bars = ±1 S.E.M.

To verify the robustness of our findings, in Experiment 3 mean global motion coherence thresholds were measured for younger and older participants for two dot densities (0.44 and 1.13 dots/deg^2^) with a constant aperture size of 113 deg^2^ (Figure 4). We (Allen et al., 2010) have previously investigated the effects of age on translational motion perception using this particular aperture size and a dot density of 0.44 dots/deg^2^ and found no difference between young and old participants when the dot contrast was relatively high (as it is in the present study). Consistent with our previous study, when the dot density was 0.44 dots/deg^2^, younger and older participants’ performance was equivalent (*t* = −1.034, *df* = 16, ns). This was also the case at the higher dot density of 1.13 dots/deg^2^ (*t* = 0.601, *df* = 16, ns).

## Discussion

The findings of the present study have shown that changes in aperture size differentially affect global motion perception in young and old participants. At larger aperture sizes (≥113 deg^2^), performance for younger and older participants was equivalent. Indeed there was no advantage for older adults at larger stimulus sizes (as might be predicted by the findings of Betts et al., 2005) nor was there an overall impairment for global motion perception. Older participants’ performance was relatively unaffected by changes in aperture size. As aperture size decreased, however, younger participants’ coherence thresholds increased (performance became worse), the result being that older participants actually exhibited superior motion perception compared to younger participants with smaller apertures. Our finding of decreases in performance with stimulus size for young participants are in agreement with those found previously by Barlow and Tripathy (1997) and the overall pattern of results was similar irrespective of whether local element density increased (Experiment 1) as aperture area decreased or remained constant (Experiment 2). The findings of Experiment 3 confirmed that the age-related differences in performance observed with small apertures was driven by image size rather than local dot density or dot number. A number of factors may contribute to the pattern of findings presented here. Each potential explanation is addressed in turn below.

### Comparison with other key studies

There were no deleterious effects of age in any of the experiments in the present study, in that even in the worst case older adults performed equivalently to younger adults with aperture areas of ≥113 deg^2^ (12 deg diameter). These findings are in agreement with those of some other studies that have used high contrast dots displaced over similar spatial extents on each frame of the motion sequence (which was 0.3 deg in the present study) and presented within similar sized regions (apertures). Snowden and Kavanagh (2006) for example found that motion coherence thresholds were unaffected by age when 400 dots were presented within a 9 deg square region (area 81 deg^2^) and displaced by 0.18 or 0.36 deg on each positional update. Arena et al. (2012) have shown that global motion perception for judging the direction of 100 dots within a 10 deg diameter aperture (area 79 deg^2^) is unaffected by age for dot displacements of 0.27 deg. Similarly, Allen et al. (2010) have shown that for high contrast dots, displaced by 0.3 deg on each frame, within a 12 deg diameter aperture (area 113 deg^2^) age does not significantly affect coherence thresholds for translational global motion.

Like the studies outlined above, Roudaia et al. (2010) have found that performance on global motion tasks is not invariably worse with aging, but rather depends on stimulus factors such as dot spatial displacement and inter-stimulus-interval. Moreover, the combination of these two factors appears to be important. They found that older adults’ performance (% correct) for identifying the direction of two-frame apparent motion was markedly worse than their younger counterparts at relatively large spatial displacements and long inter-stimulus-intervals. However the two age groups performed at equivalent levels when the dots were displaced over 0.16 and 0.32 deg between frames, but only when the inter-stimulus-interval was relatively short (0.01–0.04 s for a spatial displacement of 0.16 deg and 0.01–0.02 s for a spatial displacement of 0.32 deg). In these studies, the size of the square region in which the 300 dots appeared was 6.4 deg (area 41 deg^2^). At a similar image size (area 57 deg^2^) in the present study, we found that older adults were better than younger adults. The differences between the findings of these two studies cannot be accounted for by differences in dot number/density as we discounted this in Experiment 3. As such, we speculate that these discrepant results could arise, in principle, due to other methodological differences between the two studies. For example the dots used by Roudaia et al. (2010) were approximately 8 times smaller in spatial extent than those used in the present experiments, perhaps making it especially difficult for the older participants to resolve the individual moving elements in their display. In a similar vein, the longer motion sequence duration in the current study (eight frames compared to only two in Roudaia et al., 2010) allowed older adults more integration time to determine the global motion direction and may have benefitted performance (e.g., Barlow and Tripathy, 1997). This highlights the importance of examining how changes in specific motion sequence parameters affect motion perception in older adults, by varying them in isolation and also in conjunction with other parameters, and warrants further study.

### Spatial summation

The findings presented here could potentially, in part, reflect smaller spatial summation areas in older participants. Thresholds for older participants appeared to plateau at relatively small aperture sizes, whereas those for younger adults appeared to continue to decrease as image size increased. However, how these findings fit with the existing literature in the spatial domain is unclear. Larger, rather than smaller, summation areas for older compared to younger participants have been reported for static targets under scotopic viewing conditions (e.g., Schefrin et al., 1998). However, the majority of studies that have compared spatial summation for achromatic and chromatic static targets in young and old participants have, in the main, found no age-related differences in Ricco’s area (Dannheim and Drance, 1971; Brown et al., 1989; Redmond et al., 2010). This also appears to be the case for temporally-modulated targets (Zele et al., 2006). Drawing meaningful conclusions about the relative spatial summation areas in younger and older participants is problematic, however, both in the context of the present study and in the context of the existing literature, for a number of reasons: (1) there is no reason to assume that the findings in the spatial domain are applicable to moving stimuli; (2) measures of summation area are heavily influenced by experimental variables such as stimulus type, adaptation levels and the statistical techniques used to determine Ricco’s area, of which there are many (Redmond et al., 2010); and (3) interpreting experimental findings is difficult because, even where the “young” visual system is concerned, the physiological mechanisms underlying Ricco’s area are still unresolved (e.g., Hartline, 1940; Davila and Geisler, 1991; Swanson et al., 2004; Pan and Swanson, 2006). Finally, even if we accept that there are smaller summation areas for global motion in older adults, this does not address why their thresholds are so much lower than those of the younger participants at the smallest stimulus sizes tested.

### Cortical inhibition

We are not the first to demonstrate enhanced motion perception in older adults, relative to their younger counterparts. Betts et al. (2005) for example have shown that older adults are better than younger adults at detecting moving high contrast sine-wave gratings when image size is relatively large. It has been suggested that these findings may reflect reduced cortical inhibition in older populations (e.g., Betts et al., 2005; Bennett et al., 2007), specifically, reduced center-surround antagonism in area V5/MT (e.g., Leventhal et al., 2003; Tadin et al., 2003). With high contrast stimuli, as image size increases, more of the stimulus falls into a putative detector’s inhibitory region, reducing detection performance. For older adults, in their study, without this inhibitory region, performance continues to improve with increasing stimulus size.

This hypothesis can also be applied to our findings for global motion patterns as performance for encoding random dot motion has typically been assumed to reflect the underlying physiology of areas V1 and, particularly, area V5/MT. Direction-selective neurons in V1 encode the motion direction of local elements, the outputs of which project up-stream to area V5/MT (Movshon et al., 1985). The larger receptive fields in V5/MT integrate the local motion responses from V1 into a global representation (e.g., Livingstone et al., 2001). In our case, within this framework reduced inhibition may lead to better performance at relatively small image sizes simply due to the absence of an inhibitory surround even with smaller receptive field sizes. This assumption may not be unreasonable given that many foveal-centered receptive fields, in primate MT, sample a region of space comparable in spatial extent to the smallest aperture sizes used in the present study (e.g., Gattass and Gross, 1981; Albright and Desimone, 1987). One argument against the inhibitory surround explanation of older adults improved sensitivity with larger images is that it is, in part, mediated by baseline differences in the measure of interest such as contrast sensitivity (Aaen-Stockdale et al., 2009). For our stimuli, however, at the contrasts used, there are no significant baseline differences in our measure of interest (see Allen et al., 2010). There is good support from elsewhere for the inhibitory hypothesis. Reduced cortical inhibition in the aging primate brain has been shown to produce improved cortical function and has been linked to age-related reductions in activity of the inhibitory neurotransmitter GABA (Leventhal et al., 2003). Superior global motion perception (relative to controls) has also been shown in certain patient groups such as those suffering from schizophrenia (Tadin et al., 2006) where inhibition is known to be weak. Most recently, Tadin et al. (2011) have shown that applying repetitive transcranial magnetic stimulation (TMS) over area V5/MT can improve motion discrimination for large stimuli, an effect that they attributed to a TMS-induced weakening of surround suppression strength. As such, our findings may, at least in part, reflect reduced center-surround antagonism and hence less spatial suppression in area V5/MT in older adults.

### Neural blur

It is well known that old age leads to a contrast sensitivity loss at intermediate and high spatial frequencies, that is likely to be due mainly to neural rather than optical factors (e.g., Weale, 1975, 1986; Elliott et al., 1990). Within the context of our RDK stimulus, which necessarily contains a broad range of spatial frequencies, superior performance in older participants may reflect changes within the visual pathways that effectively result in lowpass spatial filtering of the image. The result of which may be to render the image “less dense” as higher frequency information is attenuated. There is evidence for example that the upper motion displacement threshold (D_max_) increases after lowpass spatial frequency filtering (Morgan, 1992). Furthermore, positive dioptre optical blur, which is known to degrade high spatial frequencies (Westheimer and McKee, 1980), can improve global motion perception in RDK displays (Barton et al., 1996). One notable difference between younger and older adults’ performance was that, whilst younger adults demonstrated the expected deterioration in performance at small image sizes, older adults’ performance was relatively unaffected by changes in image size. Younger adults exhibited poorest performance at the smallest image sizes. Performance improved markedly as image size initially increased and then became asymptotic at the largest aperture sizes tested. These findings may reflect the gradual encroachment of the RDK on the peripheral retina. Images in the visual periphery are effectively blurred relative to those in central vision due to high spatial frequency attenuation and hence poorer spatial acuity. Indeed, the blur-related advantage of high spatial frequency attenuation has been put forward to account for the finding that D_max_ is greater in peripheral vision (Baker and Braddick, 1985; Cleary and Braddick, 1990). In the case of older participants, if the image is blurred in the fovea as well as the periphery, performance would be unlikely to deteriorate appreciably as the image size is reduced, given that high spatial frequencies are attenuated in both the periphery and the fovea.

### Visual attention

Location-selective selective attention mechanisms operate early in visual processing (Cave and Bichot, 1999) and differences in attentional strategy and ability between older and younger adults are commonly found (e.g., Allen and Payne, 2012). Older adults are less able than their younger counterparts to extract information from a cluttered visual scene (Sekuler et al., 2000), show longer search times and larger set size effects than younger adults on visual search tasks (e.g., Madden, 2007). They are less able to divide their attention between salient environmental information and irrelevant distractors and to select a visual target whilst ignoring the distractors (e.g., Owsley et al., 1998). Older adults also show deficits on visual cueing tasks. They are slower to use cues, exhibit more errors than their younger counterparts and tend to have more difficulty distinguishing between valid and invalid cues (e.g., Hoyer and Familiant, 1987; Lincourt et al., 1997).

Changes in how older adults employ selective attention might predict changes in global motion processing with age. Indeed older adults’ global motion performance appears to be predicted by their performance on tests of selective attention, whereas this does not appear to be the case for younger adults (Mapstone et al., 2008). Our findings for global motion may reflect a more narrow field of attentional focus in older, compared to younger adults. It may in fact be advantageous for older adults to restrict their field of view to a narrower region of visual space. Vision in the periphery in older adults is particularly poor (Haegerstrom-Portnoy et al., 1999). In real terms, this means that much of the information in the visual field may be invisible and, as such, effectively useless. As a result, older adults may attend to a narrow central window as a means of improving the quality of their vision. This notion fits with the zoom lens model of visual attention which predicts that reducing the window of attention improves resolution (Eriksen and Yeh, 1985). Superior global motion perception for small display sizes may therefore reflect perceptual self learning/training in older adults in response to poor peripheral vision. This notion is consistent with recent evidence for training-related changes in the structure and function in the older adult visual system (see Lustig et al., 2009).

## Conclusions

We have shown that when the aperture within which a RDK stimulus is displayed is small, performance for determining the direction of translational global motion is markedly better in older, compared to younger, participants. These findings suggest that the normal aging process does not lead to a general decline in global motion. Indeed, in some cases, and in agreement with other studies, the normal (i.e., disease-free) aging process may be visually advantageous. As far as the neural underpinnings of our present findings are concerned, they are unlikely to be explainable simply in terms of gross differences in the spatial summation areas of younger and older participants. This is compounded by a lack of consensus in the existing literature as to what the effects of age on spatial summation actually are. In a similar vein, reduced GABA inhibition in older adults does not necessarily explain why their relative performance advantage is restricted to small image sizes. Our findings may reflect simple age-related reductions in sensitivity to high spatial frequencies but it remains to be seen if these are sufficient to account for the magnitude of the differences found at the smallest stimulus sizes. Finally, although age-related narrowing of the attentional field of view may help to explain why the older adults’ performance is largely invariant with increases in image size, it does not necessarily explain why they are so much better at the task than the younger participants with the smallest RDKs used. We are currently exploring some of these possibilities in greater detail in our laboratory.

In conclusion, this preliminary study represents somewhat surprising findings that will require further study. Indeed, in many respects they raise questions, rather than providing answers. We show that our findings cannot be explained by changes in dot density but future studies should investigate the potential interplay between image size and different RDK parameters such as signal dot trajectory, dot size (which in our study was relatively large), dot speed, spatial displacement and motion sequence duration. In doing so, to verify the robustness of the findings, testing would also ideally be conducted in a larger sample of participants.

## Conflict of interest statement

The authors declare that the research was conducted in the absence of any commercial or financial relationships that could be construed as a potential conflict of interest.

## References

[B1] Aaen-Stockdale C. R. Thompson B. Huang P. C. Hess R. F. (2009). Low-level mechanisms may contribute to paradoxical motion percepts. J. Vis. 9:9 10.1167/9.5.9 19757887

[B2] Albright T. D. Desimone R. (1987). Local precision of visuotopic organization in the middle temporal area (MT) of the macaque. Exp. Brain Res. 65, 582–592 10.1007/bf00235981 3556486

[B3] Allen H. A. Hutchinson C. V. Ledgeway T. Gayle P. (2010). The role of contrast sensitivity in global motion processing deficits in the elderly. J. Vis. 10:15 10.1167/10.10.15 20884480

[B4] Allen H. A. Payne H. (2012). Similar behaviour, different brain patterns: age-related changes in neural signatures of ignoring. Neuroimage 59, 4113–4125 10.1016/j.neuroimage.2011.10.070 22056463

[B5] Anderson S. J. Burr D. C. (1991). Spatial summation of directionally selective mechanisms in human vision. J. Opt. Soc. Am. A 8, 1330–1339 10.1364/josaa.8.001330 1919836

[B7] Arena A. Hutchinson C. V. Shimozaki S. S. (2012). The effects of age on the spatial and temporal integration of global motion. Vision Res. 58, 27–32 10.1016/j.visres.2012.02.004 22391511

[B8] Atchley P. Andersen G. J. (1998). The effect of age, retinal eccentricity and speed on the detection of optic flow components. Psychol. Aging 13, 297–308 10.1037//0882-7974.13.2.297 9640589

[B9] Baker C. L. Braddick O. J. (1985). Eccentricity-dependent scaling of the limits for short-range apparent motion perception. Vision Res. 25, 803–812 10.1016/0042-6989(85)90188-9 4024478

[B10] Barlow H. Tripathy S. P. (1997). Correspondence noise and signal pooling in the detection of coherent visual motion. J. Neurosci. 17, 7595–7966 9315913 10.1523/JNEUROSCI.17-20-07954.1997 PMC6793893

[B62] Barton J. S. Rizzo M. Nawrot N. Simpson T. (1996). Optical blur and the perception of global coherent motion in random dot cinematograms. Vision Res. 36, 3051–3059 10.1016/0042-6989(96)00063-6 8917768

[B11] Bennett P. J. Sekuler R. Sekuler A. B. (2007). The effects of aging on motion detection and direction identification. Vision Res. 47, 799–809 10.1016/j.visres.2007.01.001 17289106

[B12] Betts L. R. Taylor C. P. Sekuler A. B. Bennett P. J. (2005). Aging reduces center surround antagonism in visual motion processing. Neuron 45, 361–366 10.1016/j.neuron.2004.12.041 15694323

[B13] Billino J. Bremmer F. Gegenfurtner K. R. (2008). Differential aging of motion processing mechanisms: evidence against general perceptual decline. Vision Res. 48, 1254–1261 10.1016/j.visres.2008.02.014 18396307

[B14] Brown B. Peterken C. Bowman K. J. Crassini B. (1989). Spatial summation in young and elderly observers. Ophthalmic Physiol. Opt. 9, 310–313 10.1111/j.1475-1313.1989.tb00913.x 2622674

[B15] Cave K. R. Bichot N. P. (1999). Visuospatial attention: beyond a spotlight model. Psychon. Bull. Rev. 6, 204–223 10.3758/bf03212327 12199208

[B16] Cleary R. Braddick O. J. (1990). Masking of low frequency information in short-raneg apparent motion. Vision Res. 30, 317–327 10.1016/0042-6989(90)90046-n 2309465

[B17] Dakin S. C. Mareschal I. Bex P. J. (2005). Local and global limitations on direction integration assessed using equivalent noise. Vision Res. 45, 3027–3049 10.1016/j.visres.2005.07.037 16171844

[B18] Dannheim F. Drance S. M. (1971). Studies of spatial summation in central areas in normal people of all ages. Can. J. Ophthalmol. 6, 311–319 5125656

[B19] Davila K. D. Geisler W. S. (1991). The relative contributions of pre-neural and neural factors to areal summation in the fovea. Vision Res. 31, 1369–1380 10.1016/0042-6989(91)90058-d 1891825

[B20] Eagle R. A. Rogers B. J. (1996). Motion detection is limited by element density not spatial frequency. Vision Res. 36, 545–558 10.1016/0042-6989(96)89252-2 8855000

[B21] Edwards M. Badcock D. (1995). Global motion perception: no interaction between the first- and second-order pathways. Vision Res. 35, 2589–2602 10.1016/0042-6989(95)00003-i 7483303

[B22] Elliott D. B. Whitaker D. MacVeigh D. (1990). Neural contribution to spatiotemporal contrast sensitivity decline in healthy eyes. Vision Res. 30, 541–547 10.1016/0042-6989(90)90066-t 2339508

[B23] Eriksen C. W. Yeh Y. Y. (1985). Allocation of attention in the visual field. J. Exp. Psychol. Hum. Percept. Perform. 11, 583–597 10.1037//0096-1523.11.5.583 2932532

[B60] Folstein M. F. Robins L. N. Helzer J. E. (1983). The mini-mental state examination. Arch. Gen. Psychiatry 40, 812–812 10.1001/archpsyc.1983.01790060110016 6860082

[B24] Gattass R. Gross C. G. (1981). Visual topography of striate projection zone (MT) in posterior superior temporal sulcus of the macaque. J. Neurophysiol. 46, 621–638 7299437 10.1152/jn.1981.46.3.621

[B25] Gilmore G. C. Wenk H. E. Naylor L. A. Stuve T. A. (1992). Motion perception and aging. Psychol. Aging 7, 654–660 10.1037/0882-7974.7.4.654 1466834

[B26] Glasser D. M. Tadin D. (2010). Low-level mechanisms do not explain paradoxical motion percepts. J. Vis. 10:20 10.1167/10.4.20 20465339 PMC3098137

[B27] Haegerstrom-Portnoy G. Schneck M. E. Brabyn J. A. (1999). Seeing into old age: vision function beyond acuity. Optom. Vis. Sci. 76, 141–158 10.1097/00006324-199903000-00014 10213444

[B28] Hartline H. K. (1940). The effects of spatial summation in the retina on excitation of the fibers of the optic nerve. Am. J. Physiol. 130, 700–711

[B29] Hoyer W. J. Familiant M. E. (1987). Adult age differences in the rate of processing expectancy information. Cogn. Dev. 2, 59–70 10.1016/s0885-2014(87)90035-9

[B59] Hutchinson C. V. Arena A. Allen H. A. Ledgeway T. (2012). Psychophysical correlates of global motion processing in the aging visual system: a critical review. Neurosci. Biobehav. Rev. 36, 1266–1272 10.1016/j.neubiorev.2012.02.009 22343109

[B30] Hutchinson C. V. Ledgeway T. (2010). Spatial summation of first-order and second-order motion in human vision. Vision Res. 50, 1766–1774 10.1016/j.visres.2010.05.032 20570691

[B31] Lappin J. S. Bell H. H. (1976). The detection of coherence in moving visual patterns. Vision Res. 16, 161–168 10.1016/0042-6989(76)90093-6 1266056

[B32] Leventhal A. G. Wang Y. C. Pu M. L. Zhou Y. F. Ma Y. Y. (2003). GABA and its agonists improved visual cortical function in senescent monkeys. Science 300, 812–815 10.1126/science.1082874 12730605

[B33] Lincourt A. E. Folk C. L. Hoyer W. J. (1997). Effects of aging on voluntary and involuntary shifts of attention. Aging Neuropsychol. Cogn. 4, 290–303 10.1080/13825589708256654 29053089

[B34] Livingstone M. S. Pack C. C. Born R. T. (2001). Two-dimensional substructure of MT receptive fields. Neuron 30, 781–793 10.1016/s0896-6273(01)00313-0 11430811

[B35] Lustig C. Shah P. Seidler R. Reuter-Lorenz P. A. (2009). Ageing, training and the brain: a review and future directions. Neuropsychol. Rev. 16, 504–522 10.1007/s11065-009-9119-9 19876740 PMC3005345

[B36] Madden D. J. (2007). Aging and visual attention. Curr. Dir. Psychol. Sci. 16, 70–74 10.1111/j.1467-8721.2007.00478.x 18080001 PMC2136439

[B37] Mapstone M. Dickerson K. Duffy C. J. (2008). Distinct mechanisms of impairment in cognitive ageing and Alzheimer’s disease. Brain 131, 1618–1629 10.1093/brain/awn064 18385184 PMC13255042

[B61] Morgan M. J. (1992). Spatial filtering precedes motion detection. Nature 355, 344–346 10.1038/355344a0 1731247

[B38] Movshon J. A. Adelson E. H. Gizzi M. S. Newsome W. T. (1985). “The analysis of moving visual patterns,” in Pattern Recognition Mechanisms, ed Chagas R. G. A. C. G. C. (Rome: Vatican Press), 117–151

[B39] Owsley C. Ball K. McGwin G. Sloane M. E. Roenker D. L. White M. F. (1998). Visual processing impairment and risk of motor vehicle crash among older adults. JAMA 279, 1083–1088 10.1001/jama.279.14.1083 9546567

[B40] Pan F. Swanson W. H. (2006). A cortical pooling model of spatial summation for perimetric stimuli. J. Vis. 6:2 10.1167/6.11.2 17209726 PMC3777700

[B41] Redmond T. Zlatkova M. B. Garway-Heath D. F. Anderson R. S. (2010). The effect of age on the area of complete spatial summation for chromatic and achromatic stimuli. Invest. Ophthalmol. Vis. Sci. 51, 6533–6539 10.1167/iovs.10-5717 20671282

[B42] Roudaia E. Bennett P. J. Sekuler A. B. Pilz K. S. (2010). Spatiotemporal properties of apparanet motion perception and aging. J. Vis. 10:5 10.1167/10.14.5 21131565

[B43] Schefrin B. E. Bieber M. L. McLean R. Werner J. S. (1998). The area of complete scotopic summation enlarges with age. J. Opt. Soc. Am. A Opt. Image Sci. Vis. 15, 340–348 10.1364/josaa.15.000340 9457792

[B44] Sekuler A. B. Bennett P. J. Mamelak M. (2000). Effects of aging on the useful field of view. Exp. Aging Res. 26, 103–120 10.1080/036107300243588 10755218

[B45] Snowden R. J. Kavanagh E. (2006). Motion perception and the ageing visual system: minimum motion, motion coherence and speed discrimination thresholds. Perception 35, 9–24 10.1068/p5399 16491704

[B46] Swanson W. H. Felius J. Pan F. (2004). Perimetric defects and ganglion cell damage: interpreting linear relations using a two-stage neural model. Invest. Ophthalmol. Vis. Sci. 45, 466–472 10.1167/iovs.03-0374 14744886

[B47] Tadin D. Kim J. Doop M. L. Gibson C. Lappin J. S. Blake R. (2006). Weakened center-surround interactions in visual motion processing in schizophrenia. J. Neurosci. 2, 11403–11412 10.1523/jneurosci.2592-06.2006 17079669 PMC6674537

[B48] Tadin D. Lappin J. S. Gilroy L. A. Blake R. (2003). Perceptual consequences of centre-surround antagonism in visual motion processing. Nature 424, 312–315 10.1038/nature01800 12867982

[B49] Tadin D. Silvanto J. Pascual-Leone A. Batelli L. (2011). Improved motion perception and impaired spatial suppression following disruption of cortical area MT/V5. J. Neurosci. 31, 1279–1283 10.1523/JNEUROSCI.4121-10.2011 21273412 PMC3078722

[B50] Tran D. B. Silverman S. E. Zimmerman K. Feldon S. E. (1998). Age-related deterioration of motion perception and detection. Graefes Arch. Clin. Exp. Ophthalmol. 236, 269–273 10.1007/s004170050076 9561359

[B51] Trick G. L. Silverman S. E. (1991). Visual sensitivity to motion: age-related changes and deficits in senile dementia of the Alzheimer type. Neurology 41, 1437–1440 10.1212/wnl.41.9.1437 1891094

[B52] Watson A. B. Turano K. (1995). The optimal motion stimulus. Vision Res. 35, 325–336 10.1016/0042-6989(94)00182-l 7892728

[B53] Weale R. A. (1975). Senile changes in visual acuity. Trans. Ophthalmol. Soc. U K 95, 36–38 1064207

[B54] Weale R. A. (1986). Aging and vision. Vision Res. 26, 1507–1512 10.1016/0042-6989(86)90170-7 3303670

[B54a] Westheimer G. McKee S. P. (1980). Stereoscopic acuity with defocused and spatially filtered retinal images. J. Opt. Soc. Am. 70, 772–778 10.1364/josa.70.000772

[B55] Williams D. W. Sekuler R. (1984). Coherent global motion percepts from stochastic local motions. Vision Res. 24, 55–62 10.1016/0042-6989(84)90144-5 6695508

[B56] Wojciechowski R. Trick G. L. Steinman S. B. (1995). Topography of the age-related decline in motion sensitivity. Optom. Vis. Sci. 72, 67–74 10.1097/00006324-199502000-00005 7753530

[B57] Zele A. J. O’Loughlin R. K. Guymer R. H. Vingrys A. J. (2006). Disclosing disease mechanisms with a spatio-temporal summation paradigm. Graefes Arch. Clin. Exp. Ophthalmol. 244, 425–432 10.1007/s00417-005-0121-5 16220278

